# Evolution of *CYP2J19*, a gene involved in colour vision and red coloration in birds: positive selection in the face of conservation and pleiotropy

**DOI:** 10.1186/s12862-018-1136-y

**Published:** 2018-02-13

**Authors:** Hanlu Twyman, Staffan Andersson, Nicholas I. Mundy

**Affiliations:** 1 Department of Zoology, University of Cambridge, Cambridge, CB2 3EJ UK; 2 Department of Biological and Environmental Sciences, University of Gothenburg, 40530 Göteborg, Sweden

**Keywords:** *CYP2J19*, *CYP*, Red carotenoid coloration, Retinal oil droplets, Birds, Selection analysis, Pleiotropy

## Abstract

**Background:**

Exaggerated signals, such as brilliant colours, are usually assumed to evolve through antagonistic coevolution between senders and receivers, but the underlying genetic mechanisms are rarely known. Here we explore a recently identified “redness gene”, *CYP2J19*, that is highly interesting in this context since it encodes a carotenoid-modifying enzyme (a C4 ketolase involved in both colour signalling and colour discrimination in the red (long wavelength) spectral region.)

**Results:**

A single full-length *CYP2J19* was retrieved from 43 species out of 70 avian genomes examined, representing all major avian clades. In addition, *CYP2J19* sequences from 13 species of weaverbirds (Ploceidae), seven of which have red C4-ketocarotenoid coloration were analysed. Despite the conserved retinal function and pleiotropy of *CYP2J19*, analyses indicate that the gene has been positively selected throughout the radiation of birds, including sites within functional domains described in related *CYP* (cytochrome P450) loci. Analyses of eight further *CYP* loci across 25 species show that positive selection is common in this gene family in birds. There was no evidence for a change in selection pressure on *CYP2J19* following co-option for red coloration in the weaverbirds.

**Conclusions:**

The results presented here are consistent with an ancestral conserved function of *CYP2J19* in the pigmentation of red retinal oil droplets used for colour vision, and its subsequent co-option for red integumentary coloration. The cause of positive selection on *CYP2J19* is unclear, but may be partly related to compensatory mutations related to selection at the adjacent gene *CYP2J40.*

**Electronic supplementary material:**

The online version of this article (10.1186/s12862-018-1136-y) contains supplementary material, which is available to authorized users.

## Background

Bright colours and other exaggerated visual and acoustic displays are usually attributed to antagonistic coevolution between senders and receivers, but the genetic control of both signalling and perception are poorly known. Recently, however, a potentially widely important genetic link was found in birds between the generation and sensory discrimination of long wavelength (yellow to red) carotenoid colour hues, which are frequent targets of social and sexual selection [1–7]. Red C4-ketocarotenoid pigmentation, the main mechanism for redness in birds, was shown (in zebra finch and a hybrid canary) to depend on the gene *CYP2J19*, a member of the cytochrome P450 family of monooxygenases, which likely encodes an enzyme that catalyses the conversion of dietary yellow carotenoids into their red coloured derivatives [8, 9].

In addition to its function in coloration in some lineages, *CYP2J19* appears to be widely expressed in the avian retina [9, 10], where it is involved in colour vision by generating the C4-ketocarotenoid astaxanthin in the red oil droplets of longwave-sensitive cones [10, 11]. Like other single cone oil droplets, these act as cut-off filters that shift and narrow the bandwidths of the cone absorption spectra, predicted to lead to enhanced colour constancy and finer colour discrimination [12], in this case in the red (long wavelength) spectral region. Hence the same gene appears to function in both the generation and the detection of red colour signals. A study on the evolutionary history of *CYP2J19* in reptiles showed that it arose in the common ancestor of turtles and archosaurs [13]. The ancestral function of the gene was likely for retinal oil droplet pigmentation, with its function in red coloration being subsequently co-opted in certain avian and turtle lineages [13]. In birds, *CYP2J19* was until recently only reported in some passerines, galliforms, ostriches and cormorants [8, 9, 13–15], but given that almost all bird species have red cone oil droplets, with the exception of a few lineages such as penguins and some owls [16, 17] it is predicted that *CYP2J19* should be present and functional in most avian species. Indeed, a recent survey of avian genomes concluded that *CYP2J19* is intact in all studied taxa except certain (and notably some nocturnal) avian lineages, where it is pseudogenised [18]. Whereas most of the few birds examined to date seem to have a single *CYP2J19* gene, two copies have been reported in the zebra finch, one specialised for colour vision (*CYP2J19A*) and the other for red coloration (*CYP2J19B)* [8]. Given the importance of duplication events for functional divergence, it is an interesting question if and where *CYP1J19* duplication has occurred in other bird lineages.

Carotenoid-based coloration (yellow, orange, red) has evolved multiple times in birds in a complex pattern [19] and, within a few avian clades, the evolution of red carotenoid colour hues has been studied, notably in the genus of African widowbirds and bishops (Ploceidae; *Euplectes*) together with its sister group including the genera *Quelea* and *Foudia* [20]. In this clade, red C4-ketocarotenoid coloration evolved twice from a yellow ancestor, and is strongly associated with high hepatic (liver) expression of *CYP2J19*, whereas both yellow and red species express *CYP2J19* in the retina [21]. Moreover, in contrast to the zebra finch, *CYP2J19* occurs in a single copy in all Ploceidae studied so far [21]. Hence this is an excellent group in which to address whether the evolution of pleiotropy in *CYP2J19* through the acquisition of *CYP2J19*-based red coloration is associated with a change in the pattern of selection on *CYP2J19.*

*CYP2J19* is one member of the large family of *CYP* genes in birds that encode cytochrome P450 enzymes [14, 15]. In order to interpret the molecular evolution of *CYP2J19* we study additional *CYP* loci in multiple avian genomes, including *CYP2J40*, a gene of unknown function that lies adjacent to *CYP2J19* on chromosome 8 [8]. Previous studies on the *CYP2* family in birds have shown that positive selection is common in this family [22], but this study did not include *CYP2J19*.

The detection of selection in the genome, through the ratio of the rate of non-synonymous to synonymous mutations (dN/dS), is a powerful tool for identifying loci involved in adaptation [23], as illustrated by many compelling cases of the genetics of adaptation [24–26]. Directional selection towards a novel adaptive peak is the most common scenario invoked to explain a signature of selection at the molecular level. There is, however, another possibility, whereby the adaptive peak has already been reached but mildly deleterious alleles are still spreading through a variety of mechanisms, which leads to reduced fitness and selection for compensatory mutations that restore protein function [27]. In this scenario, positive selection is required to retain a steady state of optimal protein function. This can potentially explain how positive selection occurs in a gene with a conserved function, which might otherwise be expected to be evolving solely under purifying selection. Currently, however, there is a poor understanding of the prevalence of compensatory mutations.

In this study, we investigated the evolution of *CYP2J19* in birds, by conducting a broad analysis of avian genomes for presence, copy number and selection of *CYP2J19* with comparison to other *CYP* loci, and a focussed analysis of selection on *CYP2J19* in the weaverbirds (Ploceidae), which vary in yellow and red carotenoid coloration. More specifically, we asked (i) whether *CYP2J19* is present in all birds, (ii) what the copy number of *CYP2J19* is in different avian lineages, (iii) what the pattern of selection on *CYP2J19* across available avian genomes and how this compares to other *CYP*s, including *CYP2J40*, and (iv) whether selection on *CYP2J19* changes after it was co-opted for a pleiotropic effect on red coloration in weaverbirds.

## Methods

### Sequence acquisition

BLASTn searches were performed on 70 avian genomes in Genbank using zebra finch and chicken *CYP2J19* annotated in Ensembl 83 as query sequences [28]. The 9 exons of *CYP2J19* were blast searched individually against the 70 genomes and the resultant sequences were examined for complete open reading frames. Full-length (1431 bp) sequences of *CYP2J19* from 43 species were retained for downstream analyses (Additional file 1: Table S1). It is of interest to note that only a few of the 70 species sampled have red coloration, and only the zebra finch and American flamingo have confirmed red coloration due to ketocarotenoids.

An assembled dataset of other *CYP*s was used in a matched comparative study of *CYP* evolution (Additional file 1: Table S1). The 43 avian genomes with full-length *CYP2J19* were searched for 35 *CYP*s identified from the chicken genome, with the aim of obtaining complete data from the maximum number of *CYP*s and lineages. Orthologues from eight further full-length *CYP*s were obtained from 25 avian genomes, representing all major avian clades (Table 1). The full list of nine *CYP*s, their chromosomal locations in zebra finch, and length of ORF (open reading frame) is as follows: *CYP2J19* (Chromosome 8, 1431 bp), *CYP2J40* (Chromosome 8, 1482 bp), *CYP19A1* (Chromosome 10, 1506 bp), *CYP7A1* (Chromosome 2, 1536 bp), *CYP8B1* (Chromosome 2, 1524 bp), *CYP4V2* (Chromosome 4, 1548 bp), *CYP3A9* (Chromosome 14, 1449 bp), *CYP7B1* (Chromosome 2, 1404 bp), *CYP20A1* (Chromosome 7, 1317 bp). We also examined whether the close synteny of *CYP2J19* and *CYP2J40* on chromosome 8 in the zebra finch is conserved in other avian genomes.

**Table 1:** 70 avian genomes investigated in the study and those used in downstream analyses

Order	70 avian genomes searched	43 species with full-length *CYP2J19*	25 species used in comparative *CYP* analysis
Struthioniformes	*Struthio camelus* (African ostrich)	✓	
Apterygiformes	*Apteryx australis* (brown kiwi)		
Tinamiformes	*Tinamus guttatus* (white-throated tinamou)		
Galliformes	*Colinus virginianus* (northern bobwhite)		
	*Coturnix japonica* (Japanese quail)		
	*Gallus gallus* (red junglefowl)	✓	✓
	*Lyrurus tetrix* (black grouse)		
	*Meleagris gallopavo* (turkey)		
Anseriformes	*Anas platyrhynchos* (mallard)	✓	✓
	*Anser cygnoides* (swan goose)	✓	✓
Phoenicopteriformes	*Phoenicopterus ruber* (American flamingo)		
Podicipediformes	*Podiceps cristatus* (great-crested grebe)		
Columbiformes	*Columba livia* (rock pigeon)	✓	✓
Mesitornithiformes	*Mesitornis unicolor* (brown roatelo)	✓	
Pteroclidiformes	*Pterocles gutturalis* (yellow-throated sandgrouse)		
Apodiformes	*Calypte anna* (Anna’s hummingbird)	✓	
	*Chaetura pelagica* (chimney swift)	✓	✓
Caprimulgiformes	*Caprimulgus carolinensis* (chuck-will’s-widow)	✓	
Cuculiformes	*Cuculus canorus* (common cuckoo)	✓	✓
Otidiformes	*Chlamydotis macqueenii* (MacQueen’s bustard)		
	*Chlamydotis undulata* (houbara bustard)		
Musophagiformes	*Tauraco erythrolophus* (red-crested turaco)	✓	
Opisthocomiformes	*Opisthocomus hoazin* (hoatzin)	✓	✓
Gruiformes	*Balearica pavonina* (black crowned crane)		
	*Balearica regulorum* (grey crowned crane)	✓	✓
Charadriiformes	*Calidris pugnax* (ruff)		
	*Charadrius vociferous* (killdeer)	✓	
Gaviiformes	*Gavia stellate* (red-throated loon)		
Procellariiformes	*Fulmarus glacialis* (northern fulmar)	✓	✓
Sphenisciformes	*Aptenodytes forsteri* (emperor penguin)	✓	✓
	*Pygoscelis adeliae* (Adelie penguin)		
Suliformes	*Phalacrocorax carbo* (great cormorant)		
Pelecaniformes	*Egretta garzetta* (little egret)	✓	
	*Nipponia nippon* (crested ibis)	✓	✓
	*Pelecanus crispus* (Dalmatian pelican)		
Eurypygiformes	*Eurypyga helias* (sunbittern)		
Phaethontiformes	*Phaethon lepturus* (white-tailed tropicbird)	✓	✓
Cathartiformes	*Cathartes aura* (turkey vulture)		
Accipitriformes	*Haliaeetus albicilla* (white-tailed eagle)	✓	✓
	*Haliaeetus leucocephalus* (bald eagle)		
	*Aquila chrysaetos* (golden eagle)	✓	✓
Strigiformes	*Tyto alba* (barn owl)		
Coliiformes	*Colius striatus* (speckled mousebird)	✓	✓
Leptosomiformes	*Leptosomus discolour* (cuckoo roller)	✓	
Trogoniformes	*Apaloderma vittatum* (bar-tailed trogon)	✓	
Bucerotiformes	*Buceros rhinoceros* (rhinoceros hornbill)	✓	
Coraciiformes	*Merops nubicus* (northern carmine bee-eater)		
Piciformes	*Picoides pubescens* (downy woodpecker)	✓	✓
Cariamiformes	*Cariama cristata* (red-legged seriema)	✓	
Falconiformes	*Falco cherrug* (saker falcon)	✓	✓
	*Falco peregrinus* (peregrine falcon)	✓	✓
Psittaciformes	*Amazona aestiva* (blue-fronted amazon)		
	*Amazona vittata* (Puerto Rican parrot)		
	*Ara macao* (scarlet macaw)		
	*Melopsittacus undulatus* (budgerigar)	✓	✓
	*Nestor notabilis* (kea)	✓	
Passeriformes	*Acanthisitta chloris* (rifleman)	✓	
	*Corvus brachyrhynchos* (American crow)		
	*Corvus cornix* (hooded crow)	✓	✓
	*Ficedula albicollis* (collared flycatcher)	✓	✓
	*Geospiza fortis* (medium ground-finch)	✓	✓
	*Manacus vitellinus* (golden-collared manakin)	✓	
	*Parus major* (great tit)	✓	
	*Phylloscopus plumbeitarsus* (two-barred warbler)		
	*Pseudopodoces humilis* (Tibetan ground-tit)	✓	✓
	*Serinus canaria* (common canary)	✓	✓
	*Sturnus vulgaris* (common starling)	✓	
	*Taeniopygia guttata* (zebra finch)	✓	✓
	*Zonotrichia albicollis* (white-throated sparrow)	✓	
	*Zosterops lateralis* (silver-eye)	✓	

Full-length *CYP2J19* sequences from thirteen species of Ploceidae were used to investigate selection in relation to coloration in this clade (species with C4-ketocarotenoid coloration marked with an asterisk): *Euplectes afer*, *E. ardens, *E. axillaris,* *E. hordeaceus, *E. macroura,* *E. nigroventris, *E. orix, *Foudia madagascariensis, *Ploceus capensis*, *P. melanocephalus*, *P. velatus*, *Quelea erythrops, *Q. quelea, [19]. C4-ketocarotenoid coloration evolved independently in two clades of red ploceids: the *Foudia*/*Quelea* clade and the *E. orix/hordeaceus/nigroventris/ardens* clade [20]. The Genbank Accession numbers for all sequences used are shown in Additional file 1: Table S1.

All gene sequences were aligned in MEGA 6 [29] using MUSCLE [30]. Analyses were conducted using phylogenies obtained from BirdTree.org [31] (Additional file 2: Figures S1-S3), which combines information from multiple sources, and which for the ploceids studied here has the same topology as used in [20]. In order to check for effects of potential discordance between gene and species trees, phylogenetic reconstruction of *CYP2J19* sequences was carried out on the 43 and 25 species dataset using maximum-likelihood in PhyML-SMS (Smart Model Selection) based on Bayesian information criterion (http://www.atgc-montpellier.fr/phyml/). Evolutionary analyses were repeated for *CYP2J19* using the reconstructed gene phylogeny.

### Molecular evolution analyses

In order to investigate the presence and type of selective forces acting on *CYP* genes, the ratio of nonsynonymous to synonymous substitution rates (dN/dS = ω) was estimated using the CodeML program within PAML 4.7 [32]. Omega values less than one, equal to one, and more than one, correspond to negative, neutral and positive selection respectively. Several model comparisons were performed, including site, branch, clade and branch-site comparisons for detection of positive selection. Bonferroni correction for multiple testing was carried out in the comparative analysis of CYPs, with *n* = 9 representing the number of loci tested. All models were run several times (where applicable) applying different initial ω values in order to avoid local likelihood peaks. Likelihood ratio tests (LRT) were used to test for significance between nested models. The Bayes empirical Bayes method (BEB) was used to identify positively selected sites when significant results were found.

Site models allow the ω to vary among different codon sites [33], and comparisons were made between M1a (Nearly Neutral model) and M2a (Positive selection), and also between M7 (beta) and M8 (beta& ω) models. Model M1a allows for sites to fall into two categories: ω < 1 and ω = 1, whereas M2a includes an additional category of ω > 1. M7-M8 comparison models ω as a beta probability distribution. The M8 model has 11 site classes and includes an additional category of ω > 1 absent from M7. The critical Chi-square values for the LRT comparisons between M1a-M2a and M7-M8 were 5.99 at 5% and 9.21 at 1% under 2 degrees of freedom.

To the weaverbird *CYP2J19* dataset, we applied branch-specific models in order to assess whether the ω values varied significantly between preselected foreground and background lineages with and without C4-ketocarotenoid coloration [34]. Here, the null model (M0) assumes a single ω value across all branches. In addition, Clade model C (CmC) was applied to the weaverbird *CYP2J19* dataset in order to investigate site-specific differences in selection in relation to coloration [35]. Partitions were made based on the presence or absence of C4-ketocarotenoid coloration. Finally, branch-site models were implemented on the ploceid dataset. The modified alternative model A [36] was compared with the null model which fixes ω = 1.

## Results

BLASTn searches identified *CYP2J19*-like sequences within all of the 70 avian genomes with the exception of the bald eagle (*Haliaeetus leucocephalus*) and the two-barred warbler (*Phylloscopus plumbeitarsus*). For five species, not all 9 exons of *CYP2J19* were recovered (*Amazona aestival, Amazona vittata, Apteryx australis, Ara macao,* and *Lyrurus tetrix*), and a further 20 species were discarded due to the presence of premature stop codons. Full-length open reading frames of *CYP2J19* were obtained for the remaining 43 species, and, apart from the zebra finch, there was no evidence for duplicate copies in any of them. The synteny of *CYP2J19* and *CYP2J40* on chromosome 8 is strongly conserved in birds, and the loci were within 4 kb of each other in all cases with sufficiently long contigs (Additional file 3: Table S3).

Analysis of full-length coding sequence of *CYP2J19* in 43 species revealed significant evidence for positive selection (> 6% positively selected sites with ω > 1.3; LRS > 61.57, *p* < 0.01) under M7-M8 comparisons (Table 2). Five positively selected sites were identified by BEB, and alignment of *CYP2J19* with other avian CYP2 protein sequences described in Almeida et al. (2016) [22] showed that three of the sites (37, 122, 474) were located within predicted functional domains: Substrate Recognition Site 0 (SRS0, site 37), Heme binding domain (HEM, site 122) and Substrate Recognition Site 6 (SRS6, site 474). No evidence for positive selection was found under M1a-M2a tests (Table 2). Similar analyses using the *CYP2J19* gene phylogeny revealed similar results, with significant evidence of positive selection under M7-M8 (Additional file 4: Table S2).

**Table 2:** Site-specific analysis of *CYP2J19* for 43 avian species in PAML

Model	Parameter estimates	*lnL*	Model comparison	LRT Statistic	df	*P*-value	Positively selected sites under BEB P > 95% (bold: P > 99%)
M0	ω_0_ = 0.147	−12,990.709					
M1a	ω_0_ = 0.056 p_0_ = 0.868 ω_1_ = 1.000 p_1_ = 0.132	−12,561.580					
M2a	ω_0_ = 0.056 p_0_ = 0.868 ω_1_ = 1.000 p_1_ = 0.077 ω_2_ = 1.000 p_2_ = 0.055	−12,561.580	M1a vs. M2a	0.000	2	1.000	
M7	*p* = 0.168 q = 0.788	− 12,533.632					
M8	p_0_ = 0.935 (p_1_ = 0.065) *p* = 0.302 q = 2.898 ω_s_ = 1.383	−12,502.845	M7 vs. M8	61.574	2	0.000*	37, 122, 329, **455,** **474**

Underlined sites fall within predicted functional domains for CYP2 proteins annotated in Almeida et al. 2016 [22]

*Significant result at *p* < 0.01

Given the evidence for positive selection on *CYP2J19*, comparisons were made with other *CYP* loci. Using genomic searches, full sequences were obtained for a further eight *CYP* genes across 25 species of birds which are a subset of the 43 species with full *CYP2J19* ORFs (Table 1). Analyses revealed that six of the nine *CYPs* (*CYP2J19*, *CYP7B1*, *CYP2J40, CYP8B1, CYP4V2* and *CYP3A9*) were under significant positive site-specific selection in comparisons between M7 – M8 models, with 0.02–0.10 sites with an ω of 1.39–2.60. Of these, four *CYP*s (*CYP2J40, CYP8B1, CYP4V2* and *CYP3A9*) also showed significant site-specific selection in M1a-M2a model comparisons (Table 3).

**Table 3:** Site-specific comparisons for 9 *CYP* loci in 25 avian species

	LRT Statistic		p-value		Corrected p-value		M2a			M8		
CYP gene	M1a- M2a	M7-M8	M1a- M2a	M7-M8	M1a- M2a	M7-M8	Freq of sites with ω > 1	ω > 1	Positively selected sites under BEB *P* > 95% (bold: *P* > 99%)	Freq of sites with ω > 1	ω > 1	Positively selected sites under BEB P > 95%(bold: P > 99%)
*CYP2J19*	4.577	49.376	0.101	0.000		0.000^*^	0.018	1.944		0.083	1.387	8, 37, 233, 455
*CYP2J40*	76.397	84.911	0.000	0.000	0.000^*^	0.000^*^	0.038	2.974	56, **59**, 60**,** **61**, **80**, 247, 250, **393**	0.056	2.246	**56,** **59,** **60,** **61,** **80**, 247, 250**, 393**
*CYP19A1*	0.000	3.579	1.000	0.167			0.020	1.000		0.011	1.031	
*CYP7A1*	0.000	9.871	1.000	0.007		0.065	0.030	1.000		0.062	1.331	
*CYP8B1*	11.835	46.986	0.003	0.000	0.024^*^	0.000^*^	0.028	2.170		0.101	1.506	104
*CYP4V2*	57.130	64.122	0.000	0.000	0.000^*^	0.000^*^	0.031	3.160	31, **67, 69, 75**, 272	0.043	2.603	31, 57, **67, 69, 75**, 145, **272**
*CYP3A9*	57.980	86.997	0.000	0.000	0.000^*^	0.000^*^	0.059	2.504	**88**, 95, 135, **149**, 203, 279, 466	0.081	2.095	4, 87, **88, 95**, 135, **149**, 203, 223, **279**, 309, 466, 470
*CYP7B1*	8.154	25.295	0.017	0.000	0.153	0.000^*^	0.017	2.468		0.023	2.220	**89**, 348, 437, 458
*CYP20A1*	0.000	5.957	1.000	0.051			0.047	1.000		0.014	1.833	

Underlined sites fall within predicted functional domains for CYP2 proteins annotated in Almeida et al. 2016 [22]

*Significant result at *p* < 0.05 after *p*-value correction for multiple testing

Amino acid alignment of translated *CYP2J19* with annotated structures of CYP2 proteins showed that certain positively selected sites, identified by BEB, fall within functional domains: SRS0 (site 37) [22] and SRS-3 (site 233) [37], while positively selected sites in *CYP2J40* lie in SRS0 (sites 56, 59, 60, 61 and 80) and SRS-3 (sites 247, 250).

There was no evidence for positive selection on *CYP2J19* within the Ploceidae (Table 4). Neither was there a significant difference in ω between lineages with and those without C4-ketocarotenoid-based red coloration, either in branch models or clade models, although there was a tendency for the lineages with red C4-ketocarotenoid coloration to have a higher overall ω (Table 4). There was no indication of positive selection when applying branch-site models to lineages with red C4-ketocarotenoid coloration (Table 4).

**Table 4:** Summary of PAML results for *CYP2J19* across 13 ploceid species including site-specific, clade (“CmC”), branch and branch-site analyses

Model	Parameter estimates					*lnL*	Model comparison	LRT Statistic	df	*p*-value
M0	ω_0_ = 0.241					− 2591.727				
M1a	ω_0_ = 0.024 p_0_ = 0.782									
	ω_1_ = 1.000 p_1_ = 0.218					− 2587.623				
M2a	ω_0_ = 0.176 p_0_ = 0.985									
	ω_1_ = 1.000 p_1_ = 0.000 ω_2_ = 5.935 p_2_ = 0.015					− 2586.444	M1a vs. M2a	2.358	2	0.308
M7	*p* = 0.005 q = 0.019					−2587.657				
M8	p_0_ = 0.985 (p_1_ = 0.015)									
	*p* = 21.271 q = 99.000 ω_s_ = 5.960					− 2586.447	M7 vs. M8	2.422	2	0.298
M2a_rel ^a^	ω_0_ = 0.176 p_0_ = 0.985 ω_1_ = 1.000 p_1_ = 0.000 ω_2_ = 5.935 p_2_ = 0.015					− 2586.444				
CmC ^a^	ω_0_ = 0.091 p_0_ = 0.913 ω_1_ = 1.000 p_1_ = 0.000 ω_2_ (#0) = 1.101 p_2_ = 0.087 ω_2_ (#1) = 3.393					− 2585.570	M2a_rel vs. CmC	1.749	1	0.186
2ω branch model ^a^	ω (#0) = 0.165									
	ω (#1) = 0.370					− 2590.044	M0 vs. 2ω branch model	3.366	1	0.067
Branch-site Null ^a^	site class	0	1	2a	2b	−2586.422				
	proportion	0.677	0.144	0.148	0.031					
	background ω	0.000	1.000	0.000	1.000					
	foreground ω	0.000	1.000	1.000	1.000					
Branch-site Alternative Model A ^a^	site class	0	1	2a	2b	−2586.295	Null vs. Alternative Model A	0.255	1	0.613
	proportion	0.756	0.147	0.081	0.016					
	background ω	0.012	1.000	0.012	1.000					
	foreground ω	0.012	1.000	1.939	1.939					

^a^Branch partitions for Clade model C, Branch and Branch-site models: #1 = the two clades containing ketocarotenoid species: (*E. orix*, *E. hordeaceus*, *E. nigroventris* and *E. ardens*) and (*F. madagascariensis, Q. erythrops* and *Q. quelea*); #0 = all other lineages

## Discussion

A single copy of *CYP2J19* was identified in a phylogenetically broad range of avian lineages, supporting a single *CYP2J19* gene as the ancestral avian state. Considering that red C4-ketocarotenoid coloration has a patchy distribution in birds (including the lineages sampled here), whereas red retinal oil droplets are present in almost all birds examined, the contribution to colour vision is the ancestral and likely strongly conserved function of *CYP2J19.* The finding of positive rather than purifying selection on *CYP2J19* across all lineages analysed is thus somewhat surprising. There was no evidence that the acquisition of a second, likely sexually-selected, function in red coloration affected the selective pressure on *CYP2J19*. However, there was evidence for positive selection on *CYP2J40*, a syntenic gene to *CYP2J19* of unknown function, which may plausibly lead to the evolution of compensatory mutations in *CYP2J19.*

In most avian species examined, there was evidence for the presence of *CYP2J19*. This concords with a recent independent study on the evolution of *CYP2J19* in birds [18]. Although full-length open reading frames were only retrieved for 43/70 (61%) of species, these 43 species span most major avian lineages and it seems likely that poor genome quality can account for much of the missing data. Species without a full length *CYP2J19* ORF in our study include the barn owl and the southern brown kiwi that were identified in [16] based on retinal transcriptome data as likely true pseudogenization events reflecting a nocturnal lifestyle. Of the 43 species with intact ORFs, only the zebra finch (*Taeniopygia guttata*) has been confirmed to possess red C4-ketocarotenoid based coloration [1, 38]. By contrast, it is thought that almost all birds have red oil droplets in their retinas, including some nocturnal lineages such as the tawny owl [39]. Hence, the results presented here support the notion that *CYP2J19* has an ancestral, conserved function in avian colour vision and its function in red coloration appears to have been independently gained along specific bird lineages. This would most likely have been through changes in the patterns of expression of *CYP2J19* as a result of in *cis*-regulatory sequences and/or *trans*-acting factors. This concords with previous findings investigating the origin of *CYP2J19* in the reptiles [13].

Analyses of the 43 species dataset as well as the reduced 25 species dataset consistently revealed that positive selection acted on *CYP2J19* throughout its evolution in birds. In particular, site-specific selection was found to act within the newly defined substrate recognition site (SRS0) of *CYP2J19* for both datasets [22]. This is a novel finding and somewhat surprising given the inferred conserved function of *CYP2J19* in the retina, where it is involved in generating the C4-ketocarotenoid astaxanthin that is the major component of all red cone oil droplets examined [10]. The co-option of *CYP2J19* for a function in red coloration might provide a further constraint on *CYP2J19* evolution, or alternatively might necessitate adjustments for this second function. However, no evidence was found for a change in mode of selection on *CYP2J19* in ploceids, where a direct comparison can be made between lineages with exaggerated red C4-ketocarotenoid coloration and those without.

Extending the analyses to eight other *CYP* loci showed that selection is common in the gene family. Five of the loci showed evidence for positive selection and these included genes that have been shown in mammals to be involved in bile acid synthesis (oxysterol 7α-hydroxylase (*CYP7B1*) and sterol 12α -hydroxylase (*CYP8B1*)) [40–43], fatty-acid oxidation (fatty acid ω-hydroxylase, *CYP4V2*) [44] and control of sexual dimorphism (*CYP3A9*) [45–48]. The remaining gene under positive selection is *CYP2J40*, of unknown function. This gene is of particular interest since it lies adjacent to *CYP2J19* on chromosome eight in zebra finch. It has one of the strongest signatures of positive selection among the loci studied, and, consistent with previous results (where the gene is named *CYP2J_2* [22]), several positively selected sites lie in the functional domain SRS0. The three *CYP* loci found not to be under positive selection are: the aromatase, *CYP19A1*, which plays a crucial role in sex determination, female receptivity and social interactions, and appears to be well conserved in nearly all species studied [49–53]; *CYP7A1*, which catalyses the first step in bile acid synthesis [54, 55]; and *CYP20A1*, of unknown function.

Overall, therefore, although certain loci with well-conserved functions (notably *CYP19A1*) do not show evidence of positive selection, there are many *CYP* loci which do show evidence of positive selection across a broad phylogenetic sampling of birds. Moreover, the sample of 9 *CYP* loci are likely among the most conserved in the avian genome since only identifiable *CYP* genes were targeted in diverse genomes. A study focusing on the *CYP2* gene family, which is believed to primarily be involved in metabolising toxins, also found evidence for widespread positive selection (6 out of 12 *CYP2* subfamilies, and 11 out of 17 loci; Almeida et al. 2016 [22]). The results presented here are thus not unusual for *CYP* loci in general, but while the selection of many of them may be attributed to coevolution with e.g. new toxins, the question remains why some *CYP* loci with more conserved functions, such as *CYP2J19,* are also evolving under positive selection. For *CYP2J19,* an intriguing possibility is that it is affected by selection acting on *CYP2J40*, which is within 4 kb of *CYP2J40* on chromosome 8 in all lineages for which there is sufficient genomic information (Additional file 3). Specifically, it could be that directional selection on *CYP2J40* sometimes leads to fixation of linked mildly deleterious alleles at *CYP2J19* that are then followed by positive selection for compensatory mutations. Establishing the function of *CYP2J40* would be helpful in assessing this.

## Conclusion

To conclude, a single copy of *CYP2J19* was found to be widespread across avian lineages, which is consistent with a conserved ancestral function in colour vision and subsequent co-option for red integumentary coloration. Like several other *CYP* loci, including some with conserved functions, *CYP2J19* shows evidence of evolving under positive selection across birds. The cause of the positive selection on *CYP2J19* is unclear. There is no evidence for a change in selection pressure on *CYP2J19* following co-option for red coloration, but one factor may be compensatory mutations related to selection at the adjacent gene *CYP2J40.*

## Additional files

Additional file 1: Accession numbers of sequences studied. (DOCX 58 kb)

Additional file 2: Species phylogenies used for PAML analyses. (DOCX 94 kb)

Additional file 3: Intergenic distances between *CYP2J19* and *CYP2J40* in avian genomes. (DOCX 54 kb)

Additional file 4: Results for analysis of selection on *CYP2J19* using gene tree versus species tree. (DOCX 19 kb)

## Abbreviations

BEB: Bayes empirical Bayes
*CYP*: Gene encoding a cytochrome P450 enzyme
dN: Rate of non-synonymous substitution
dS: Rate of synonymous substitution
HEM: Heme binding domain
LRT: Likelihood ratio test
ORF: Open reading frame
SRS: Substrate recognition domain
