# Supplementary material for: Evolution of CYP2J19, a gene involved in colour vision and red coloration in birds: positive selection in the face of conservation and pleiotropy
Source: BMC Evol Biol. 2018 Feb 13;18:22. doi: 10.1186/s12862-018-1136-y (PMC5812113; doi:10.1186/s12862-018-1136-y)
Supplement: Supplementary file 1 — Accession numbers of sequences studied. (DOCX 58 kb) [file 12862_2018_1136_MOESM1_ESM.docx]

**Additional file 1: Table S1.** Genbank accessions for sequences utilised in this study, including *CYP2J19* from genomes of 43 avian species and 13 ploceid species (marked with an asterisk), and the 25-species dataset of *CYP2J40, CYP3A9, CYP4V2, CYP7A1, CYP7B1, CYP8B1, CYP19A1,* and *CYP20A.*

| Species | *CYP2J19* | *CYP2J40* | *CYP3A9* | *CYP4V2* | *CYP7A1* | *CYP7B1* | *CYP8B1* | *CYP19A1* | *CYP20A1* |
| --- | --- | --- | --- | --- | --- | --- | --- | --- | --- |
| *Acanthisitta chloris* | XM_009079826.1 |  |  |  |  |  |  |  |  |
| *Anas platyrhynchos* | XM_021274710.1 | XM_021274738.1 | XM_005020119.2 | XM_005008977.2 | NM_001310351.1 | XM_013096093.2 | XM_021275226.1 | XM_013106596.2 | XM_021270472.1 |
| *Anser cygnoides* | XM_013176140.1 | XM_013176437.1 | XM_005020119.2 | XM_013179930.1 | XM_013185866.1 | XM_013185907.1 | XM_013182375.1 | XM_013177527.1 | XM_013171164.1 |
| *Apaloderma vittatum* | XM_009877501.1 |  |  |  |  |  |  |  |  |
| *Aptenodytes forsteri* | XM_009287508.2 | XM_009287413.1 | XM_009286405.1 | XM_009277380.2 | XM_009289291.2 | XM_019473652.1 | XM_009275950.2 | XM_009276478.2 | XM_019474689.1 |
| *Aquila chrysaetos* | XM_011572675.1 | XM_011572106.1 | XM_011579193.1 | XM_011578961.1 | XM_011590546.1 | XM_011590595.1 | XM_011573166.1 | XM_011592869.1 | XM_011577704.1 |
| *Balearica regulorum* | XM_010298704.1 | XM_010298703.1 | XM_010308455.1 | XM_010299974.1 | XM_010309583.1 | XM_010299709.1 | XM_010300461.1 | XM_010302939.1 | XM_010313079.1 |
| *Buceros rhinoceros* | XM_010134868.1 |  |  |  |  |  |  |  |  |
| *Calypte anna* | XM_008500861.1 |  |  |  |  |  |  |  |  |
| *Caprimulgus carolinensis* | XM_010171344.1 |  |  |  |  |  |  |  |  |
| *Cariama cristata* | XM_009697696.1 |  |  |  |  |  |  |  |  |
| *Chaetura pelagica* | XM_009993611.1 | XM_009993579.1 | XM_009993683.1 | XM_010002468.1 | XM_009995531.1 | XM_009995544.1 | XM_009994541.1 | XM_009994442.1 | XM_009996334.1 |
| *Charadrius vociferus* | XM_009884012.1 |  |  |  |  |  |  |  |  |
| *Colius striatus* | XM_010207702.1 | XM_010207701.1 | XM_010210441.1 | XM_010210805.1 | XM_010197826.1 | XM_010205842.1 | XM_010201258.1 | XM_010208731.1 | XM_010205731.1 |
| *Columba livia* | XM_021295744.1 | XM_005498904.2 | XM_005504507.1 | XM_013371895.2 | XM_005506490.2 | XM_021292967.1 | XM_005510619.3 | XM_021297872.1 | XM_021289782.1 |
| *Corvus cornix* | XM_010408755.3 | XM_010408754.3 | XM_010392382.3 | XM_010401571.3 | XM_010401149.3 | XM_019289246.2 | XM_010394769.2 | XM_010391171.3 | XM_019290584.1 |
| *Cuculus canorus* | XM_009569206.1 | XM_009569166.1 | XM_009564589.1 | XM_009565655.1 | XM_009563286.1 | XM_009570940.1 | XM_009558263.1 | XM_009555977.1 | XM_009559796.1 |
| *Egretta garzetta* | XM_009636169.1 |  |  |  |  |  |  |  |  |
| **Euplectes afer* | MG255081 |  |  |  |  |  |  |  |  |
| **Euplectes ardens* | MG255079 |  |  |  |  |  |  |  |  |
| **Euplectes axillaris* | MG255076 |  |  |  |  |  |  |  |  |
| **Euplectes hordeaceus* | MG255082 |  |  |  |  |  |  |  |  |
| **Euplectes macroura* | MG255080 |  |  |  |  |  |  |  |  |
| **Euplectes nigroventris* | MG255078 |  |  |  |  |  |  |  |  |
| **Euplectes orix* | MG255077 |  |  |  |  |  |  |  |  |
| *Falco cherrug* | XM_005440438.1 | XM_005440387.1 | XM_005435645.2 | XM_005433775.1 | XM_005443218.1 | XM_005443207.1 | XM_005442321.1 | XM_014279568.1 | XM_014282931.1 |
| *Falco peregrinus* | XM_005241546.1 | XM_005241526.1 | XM_005228984.2 | XM_005243142.1 | XM_005238225.1 | XM_005231540.1 | XM_005229542.1 | XM_013302440.1 | XM_005237063.1 |
| *Ficedula albicollis* | XM_005050670.2 | XM_005050607.2 | XM_005054427.2 | XM_005045156.2 | XM_005042095.1 | XM_016296773.1 | XM_005040499.2 | XM_016300857.1 | XM_005049123.2 |
| **Foudia madagascariensis* | MG255083 |  |  |  |  |  |  |  |  |
| *Fulmarus glacialis* | XM_009578787.1 | XM_009578786.1 | XM_009580983.1 | XM_009582248.1; XM_009584296.1 | XM_009580105.1 | XM_009582121.1 | XM_009582839.1 | XM_009576566.1 | XM_009579072.1 |
| *Gallus gallus* | XM_422553.4 | NM_001329480.1 | NM_001001751.2 | NM_001001879.1 | NM_001001753.1 | XM_015282719.1 | NM_001005571.1 | NM_001001761.2 | XM_426572.5 |
| *Geospiza fortis* | XM_005423013.1 | XM_005422968.1 | XM_005420346.1 | XM_005415515.1 | XM_005418329.1 | XM_014307867.1 | XM_014309990.1 | XM_005419646.1 | XM_005415905.1 |
| *Haliaeetus albicilla* | XM_009918785.1 | XM_009918784.1 | XM_009917758.1 | XM_009926412.1; XM_009915203.1 | XM_009929634.1 | XM_009923473.1 | XM_009930632.1 | XM_009918654.1 | XM_009928676.1; XM_009926990.1 |
| *Leptosomus discolor* | XM_009959501.1 |  |  |  |  |  |  |  |  |
| *Manacus vitellinus* | XM_008925215.2 |  |  |  |  |  |  |  |  |
| *Melopsittacus undulatus* | XM_005151299.2 | XM_005151167.1 | XM_005145259.2 | XM_005149054.1 | XM_005152525.1 | XM_005152514.1 | XM_005150004.2 | XM_013128246.1 | XM_005144666.1 |
| *Mesitornis unicolor* | XM_010180413.1 |  |  |  |  |  |  |  |  |
| *Nestor notabilis* | XM_010016308.1 |  |  |  |  |  |  |  |  |
| *Nipponia nippon* | XM_009461316.1 | XM_009461226.1 | XM_009467924.1 | XM_009474838.1 | XM_009473050.1 | XM_009461635.1 | XM_009475332.1 | XM_009470145.1 | XM_009462313.1 |
| *Opisthocomus hoazin* | XM_009932983.1 | XM_009932970.1 | XM_009936496.1 | XM_009940190.1 | XM_009937707.1 | XM_009937720.1 | XM_009931017.1 | XM_009940236.1 | XM_009945853.1 |
| *Parus major* | XM_015636452.2 |  |  |  |  |  |  |  |  |
| *Phaethon lepturus* | XM_010290454.1 | XM_010290451.1 | XM_010288127.1 | XM_010291815.1 | XM_010292189.1 | XM_010294667.1 | XM_010285332.1 | XM_010284818.1 | XM_010294168.1 |
| *Picoides pubescens* | XM_009910004.1 | XM_009902172.1 | XM_009896989.1 | XM_009902218.1 | XM_009904105.1 | XM_009904140.1 | XM_009898147.1 | XM_009897237.1 | XM_009899065.1 |
| **Ploceus capensis* | MG255085 |  |  |  |  |  |  |  |  |
| **Ploceus melanocephalus* | MG255084 |  |  |  |  |  |  |  |  |
| **Ploceus velatus* | MG255086 |  |  |  |  |  |  |  |  |
| *Pseudopodoces humilis* | XM_005521306.2 | XM_014250791.1 | XM_005523013.2 | XM_005517680.2 | XM_005517137.2 | XM_014256646.1 | XM_014256772.1 | XM_005521497.1 | XM_005519866.2 |
| **Quelea erythrops* | MG255075 |  |  |  |  |  |  |  |  |
| **Quelea quelea* | MG255074 |  |  |  |  |  |  |  |  |
| *Serinus canaria* | XM_009088928.2 | XM_009088772.2 | XM_009092083.2 | XM_009100726.2 | XM_018910372.1 | XM_009085769.2 | XM_009085759.2 | XM_009089921.2 | XM_009088050.2 |
| *Struthio camelus* | XM_009689172.1 |  |  |  |  |  |  |  |  |
| *Sturnus vulgaris* | XM_014870671.1 |  |  |  |  |  |  |  |  |
| *Taeniopygia guttata* | XM_002190498.2 | XM_002198481.2 | XM_002190672.2 | XM_002191035.3 | XM_002198299.3 | XM_012572087.1 | XM_002192960.2 | NM_001076691.2 | XM_002197409.3 |
| *Tauraco erythrolophus* | XM_009984196.1 |  |  |  |  |  |  |  |  |
| *Zonotrichia albicollis* | XM_005492302.2 |  |  |  |  |  |  |  |  |
| *Zosterops lateralis* | LAII01000109.1 |  |  |  |  |  |  |  |  |
