# Supplementary material for: Evolution of CYP2J19, a gene involved in colour vision and red coloration in birds: positive selection in the face of conservation and pleiotropy
Source: BMC Evol Biol. 2018 Feb 13;18:22. doi: 10.1186/s12862-018-1136-y (PMC5812113; doi:10.1186/s12862-018-1136-y)
Supplement: Supplementary file 2 — Species phylogenies used for PAML analyses. (DOCX 94 kb) [file 12862_2018_1136_MOESM2_ESM.docx]

**
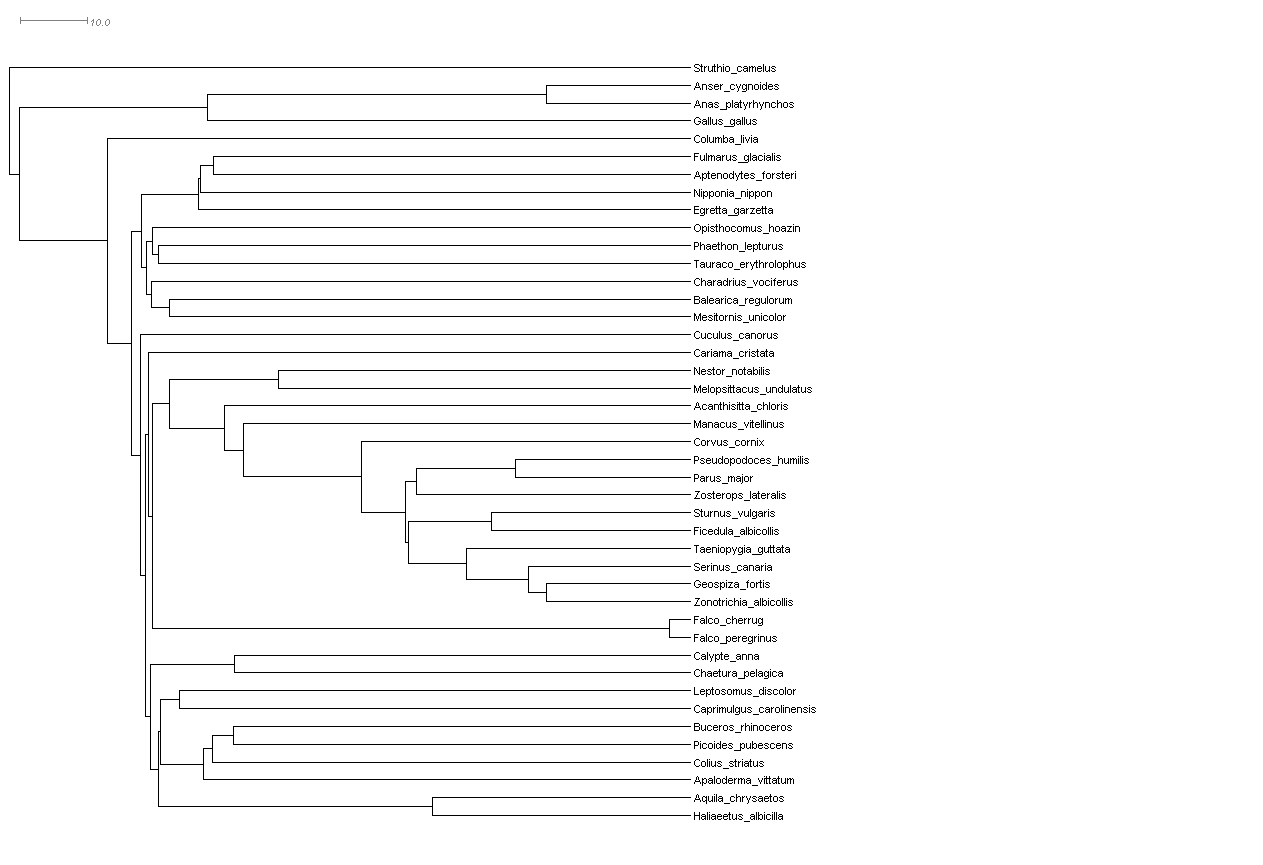
Additional file 2**

Figure S1. Phylogeny of all 43 species used in the study taken from BirdTree.org (Jetz *et al.* 2012)


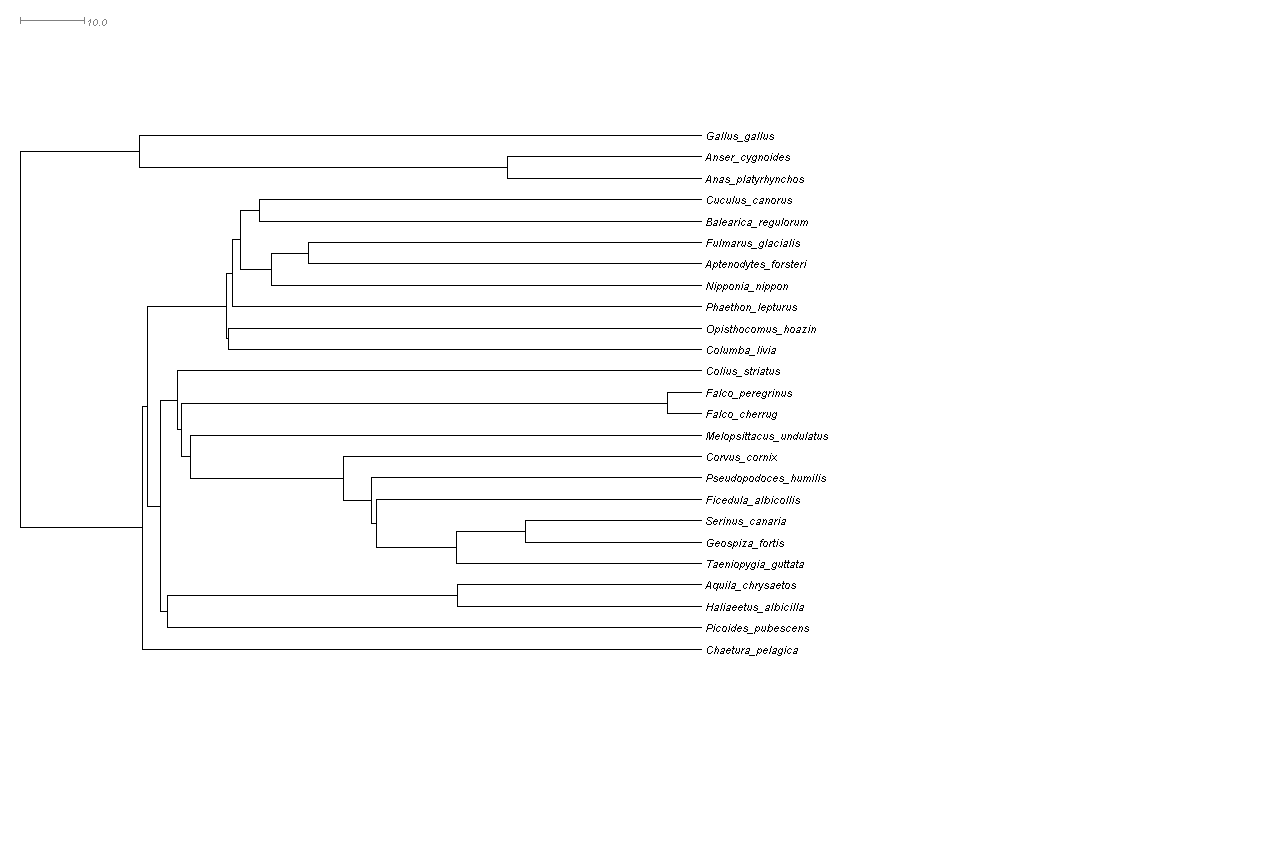


Figure S2. Phylogeny of 25 species used in the matched comparative analysis of *CYP* genes taken from BirdTree.org (Jetz *et al.* 2012)


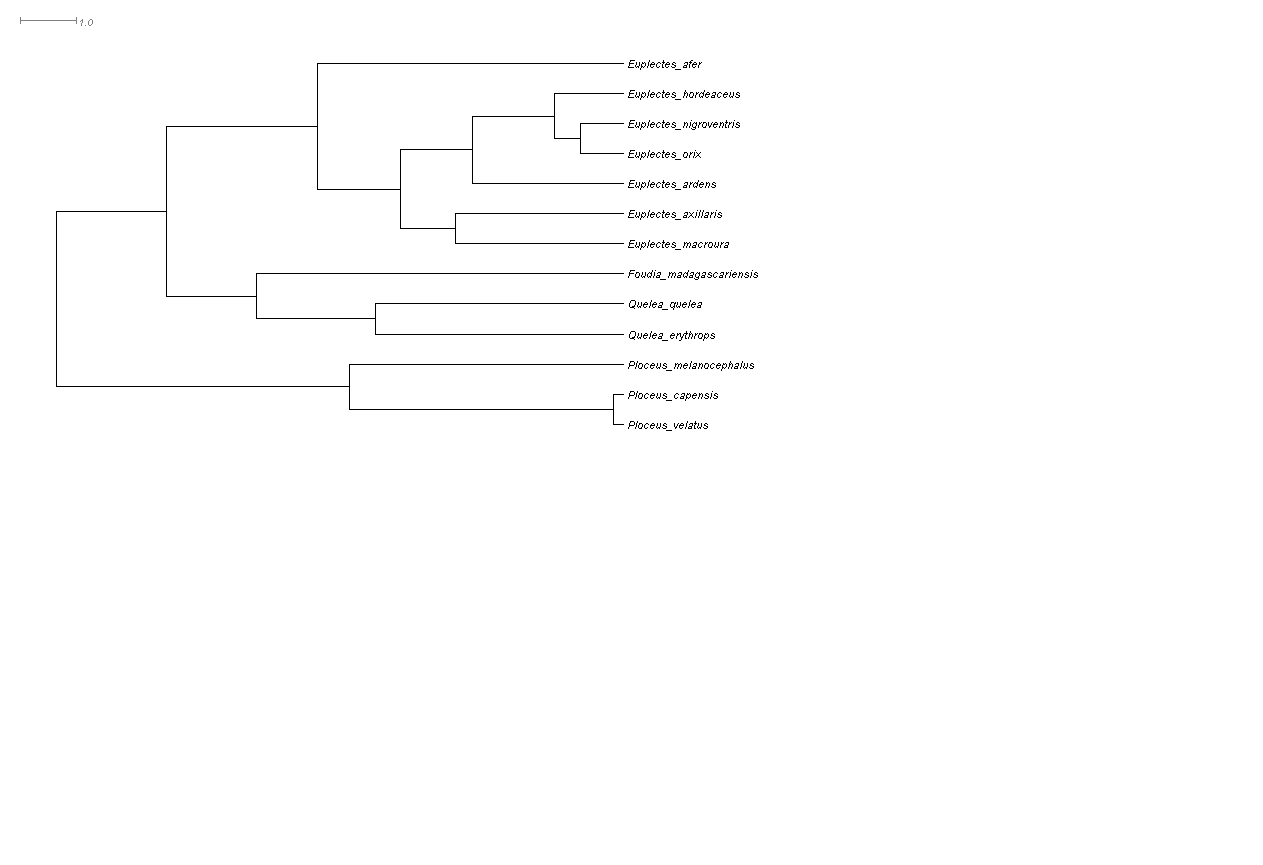
Figure S3. Phylogeny of 13 ploceid species taken from BirdTree.org (Jetz *et al.* 2012)
