# Supplementary material for: Evolution of CYP2J19, a gene involved in colour vision and red coloration in birds: positive selection in the face of conservation and pleiotropy
Source: BMC Evol Biol. 2018 Feb 13;18:22. doi: 10.1186/s12862-018-1136-y (PMC5812113; doi:10.1186/s12862-018-1136-y)
Supplement: Supplementary file 3 — Intergenic distances between CYP2J19 and CYP2J40 in avian genomes. (DOCX 54 kb) [file 12862_2018_1136_MOESM3_ESM.docx]

**Additional file 3**

Table S3. Genomic distances between *CYP2J19* and *CYP2J40* (end of exon 9 of *CYP2J19* to beginning of exon 1 of *CYP2J40*) for the 43 species with *CYP2J19* ORFs. Genomes where intergenic distances were unavailable (*CYP2J19* and *CYP2J40* on different contigs) are shown by dashes.

| Species | Intergenic distances between *CYP2J19* and *CYP2J40* (bp) |
| --- | --- |
| *Acanthisitta chloris* | - |
| *Anas platyrhynchos* | 2,218 |
| *Anser cygnoides* | 2,382 |
| *Apaloderma vittatum* | - |
| *Aptenodytes forsteri* | - |
| *Aquila chrysaetos* | 2,893 |
| *Balearica regulorum* | 3,473 |
| *Buceros rhinoceros* | - |
| *Calypte anna* | 3,034 |
| *Caprimulgus carolinensis* | - |
| *Cariama cristata* | - |
| *Chaetura pelagica* | 2,879 |
| *Charadrius vociferus* | - |
| *Colius striatus* | 4,162 |
| *Columba livia* | - |
| *Corvus cornix* | 2,240 |
| *Cuculus canorus* | 3,258 |
| *Egretta garzetta* | 3,537 |
| *Falco cherrug* | 3,414 |
| *Falco peregrinus* | 3,428 |
| *Ficedula albicollis* | 3,946 |
| *Fulmarus glacialis* | 3,476 |
| *Gallus gallus* | 1,277 |
| *Geospiza fortis* | 3,568 |
| *Haliaeetus albicilla* | 3,674 |
| *Leptosomus discolor* | 3,103 |
| *Manacus vitellinus* | 2,441 |
| *Melopsittacus undulatus* | 2,628 |
| *Mesitornis unicolor* | - |
| *Nestor notabilis* | - |
| *Nipponia nippon* | 3,502 |
| *Opisthocomus hoazin* | 3,469 |
| *Parus major* | 3,519 |
| *Phaethon lepturus* | 3,463 |
| *Picoides pubescens* | - |
| *Pseudopodoces humilis* | 3,522 |
| *Serinus canaria* | 749 |
| *Struthio camelus* | - |
| *Sturnus vulgaris* | 3,065 |
| *Taeniopygia guttata* | 2,561 |
| *Tauraco erythrolophus* | - |
| *Zonotrichia albicollis* | - |
| *Zosterops lateralis* | - |
