# Supplementary material for: Evolution of CYP2J19, a gene involved in colour vision and red coloration in birds: positive selection in the face of conservation and pleiotropy
Source: BMC Evol Biol. 2018 Feb 13;18:22. doi: 10.1186/s12862-018-1136-y (PMC5812113; doi:10.1186/s12862-018-1136-y)
Supplement: Supplementary file 4 — Results for analysis of selection on CYP2J19 using gene tree versus species tree. (DOCX 19 kb) [file 12862_2018_1136_MOESM4_ESM.docx]

| Gene | N | Phylogeny used | LRT Statistic | | p-value | | M2a | | | M8 | | |
| --- | --- | --- | --- | --- | --- | --- | --- | --- | --- | --- | --- | --- |
|  |  |  | **M1a- M2a** | **M7-M8** | **M1a- M2a** | **M7-M8** | **Freq of sites with ω>1** | **ω>1** | **Positively selected sites under BEB P>95%**  **(bold: P>99%)** | **Freq of sites with ω>1** | **ω>1** | **Positively selected sites under BEB P>95%**  **(bold: P>99%)** |
| *CYP2J19* | 43 | Species tree⬥ | 0.000 | 61.574 | 1.000 | 0.000* | - | - | - | 0.065 | 1.383 | 37, 122, 329, **455, 474** |
| *CYP2J19* | 43 | Gene tree† | 9.212 | 55.431 | 0.010* | 0.000* | 0.012 | 2.222 | 455 | 0.063 | 1.367 | 8, 122, **455**, 474 |
| *CYP2J19* | 25 | Species tree⬥ | 4.577 | 49.376 | 0.101 | 0.000* | 0.018 | 1.944 | - | 0.083 | 1.387 | 8, 37, 233, 455 |
| *CYP2J19* | 25 | Gene tree† | 3.957 | 43.148 | 0.138 | 0.000* | 0.014 | 2.027 | - | 0.479 | 1.382 | 8, 455 |

**Additional file 4**

Table S2. Site-specific model results in PAML for *CYP2J19* using the species tree and *CYP2J19* gene tree

⬥See Figures S1 and S2.

†Phylogenetic reconstructions were performed using maximum-likelihood in PhyML-SMS (Smart Model Selection) based on Bayesian information criterion (<http://www.atgc-montpellier.fr/phyml/>). Selected model for 43 species: K80 + G4 (0.678) + I (0.369), and for 25 species: K80 + G4 (0.671) + I (0.360).

*Significant result at p < 0.05.

Underlined sites fall within predicted functional domains for CYP2 proteins annotated in Almeida *et al.* 2016.
